# Chylous Ascites, a Rare Adverse Reaction to Selpercatinib

**DOI:** 10.7759/cureus.95944

**Published:** 2025-11-02

**Authors:** Adriana Dias, Joao O Pereira, Daniela Madama

**Affiliations:** 1 Pulmonology Department, Unidade Local de Saúde de Coimbra, Coimbra, PRT

**Keywords:** chylous ascites, lung cancer, ret mutations, selpercatinib, tyrosine kinase inhibitor

## Abstract

Selpercatinib improves outcomes in Rearranged during Transfection (RET) fusion-positive non-small cell lung cancer (NSCLC) but can rarely cause chylous ascites (CA). We report the case of a 53-year-old man with recurrent RET-positive lung adenocarcinoma who developed CA 17 months after starting selpercatinib. Reducing the selpercatinib dose and implementing a low long-chain triglyceride diet led to symptom relief, stable ascites, and maintained tumor response. This case highlights effective management of tyrosine kinase inhibitor (TKI)-induced CA without compromising cancer control.

## Introduction

Tyrosine kinase inhibitors (TKIs) have transformed the treatment of non-small cell lung cancer (NSCLC), especially in cases driven by specific genetic mutations [1,2]. Rearranged during Transfection (RET) is a proto-oncogene located on chromosome 10q11.2 that encodes a receptor tyrosine kinase necessary for cell survival, proliferation, migration, and differentiation [3]. Aberrant activation of RET occurs through gene fusions or point mutations, mechanisms that drive oncogenesis in several cancer types. In NSCLC, particularly lung adenocarcinoma, RET fusions are identified in approximately 1-2% of patients, most frequently among younger, non-smoking individuals. The most common fusion partner is kinesin family member 5B (KIF5B), followed by coiled-coil domain containing 6 (CCDC6) and nuclear receptor coactivator 4 (NCOA4). Although RET point mutations are rare in NSCLC, they are a well-established driver in medullary thyroid carcinoma [3].

Selpercatinib is a next-generation targeted therapy designed to inhibit RET fusions and mutations, used in advanced or metastatic RET fusion-positive NSCLC, with progression-free survival benefit, as shown in the clinical trial LIBRETTO-001 [1,4]. In this trial, 105 patients with advanced NSCLC, previously treated with platinum-based chemotherapy, received treatment with selpercatinib, with an objective response (complete or partial) in 70% of the cases. Despite these advances, rare but significant adverse events have emerged, including chylous effusions such as chylothorax and chylous ascites (CA), with recent multicenter studies reporting an incidence up to 7% in patients treated with TKIs [5].

Given the rarity and diagnostic challenges of CA in this clinical setting, evidence regarding the underlying pathophysiology and management strategies remain scarce. Here, we describe the case of a patient with advanced RET-fusion positive NSCLC who developed CA during selpercatinib therapy, emphasizing the importance of recognizing this uncommon treatment-related event.

## Case presentation

A 53-year-old male patient (non-smoker) with no significant previous medical history was diagnosed with early-stage (T2bN0M0) lung adenocarcinoma in 2018. He underwent right upper lobectomy and lymphadenectomy in 2018, without surgical complications. Due to visceral pleura invasion on histopathological examination of the resected specimen (pT2b, R0, L0), the patient received four cycles of adjuvant chemotherapy with cisplatin plus vinorelbine. During follow-up, in 2021, suspicious unilateral pulmonary nodules and increased pleural thickening were detected on chest computed tomography (CT), without pleural effusion. A right inferior lobe nodule with a 14 mm diameter was biopsied, confirming the recurrence of lung adenocarcinoma. Next-generation sequencing was performed using the tissue obtained from the biopsy, and it identified a RET fusion in the gene KIF5B. After multidisciplinary discussion, the patient was initiated on selpercatinib 160 mg, twice daily. A partial response was observed, according to the Response Evaluation Criteria in Solid Tumors (RECIST) version 1.1 criteria [6], at three, six, and 12 months. But 17 months after treatment initiation, the patient developed progressive abdominal pain and distension as well as changes in bowel habits.

**Figure 1:**
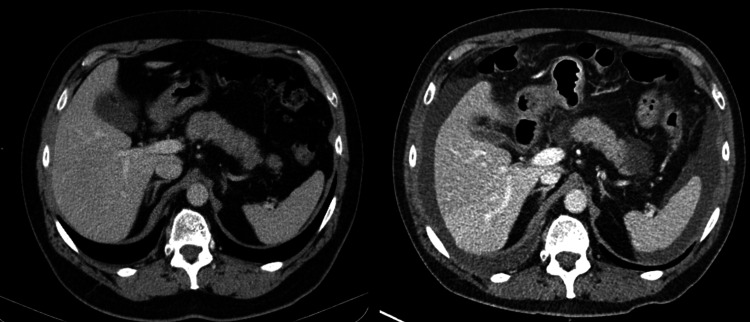
Abdominal CT Shows absence of peritoneal effusion (left) before selpercatinib treatment and moderate peritoneal effusion, mainly peri-hepatic and peri-splenic (right), 17 months after selpercatinib initiation.

A thoraco-abdominal CT showed a new onset moderate peritoneal effusion, as shown in Figure 1, without associated pleural effusion.

Endoscopy and colonoscopy were unremarkable. Abdominal ultrasound showed peritoneal effusion in all quadrants of the abdomen and diagnostic paracentesis was performed with the drainage of 20 mL of milky fluid. The peritoneal fluid analysis is summarized in Table 1 and was biochemically classified as CA (triglyceride concentration of 6879 mg/dL).

**Table 1:** Characteristics of the patient's ascitic fluid and reference values [7]

Characteristics	Patient's values	Chylous ascites reference values
Color	Milky and cloudy	Milky and cloudy
Triglyceride level	6879 mg/dL	Above 200 mg/dL
Cell count	2818 cel/uL	Above 500 cel/uL
Serum Ascites Albumin Gradient (SAAG)	2,1 g/dL	Depends on the underlying etiology
Culture	Negative for bacterial growth	Depends on the underlying etiology
Cytology	Negative for malignant cells	Depends on the underlying etiology

The peritoneal fluid culture was negative and cytological examination revealed reactive cells with no evidence of malignancy.

A clinical suspicion of Common Terminology Criteria for Adverse Events (CTCAE) [8] grade 2 selpercatinib-induced CA was thus raised and the dose of selpercatinib was reduced to 80 mg twice daily. Dietary advice of a diet low on long-chain triglycerides was given. The patient showed symptomatic improvement, with resolution of abdominal pain and normalization of bowel habits. Although ascites persisted on follow-up thoracoabdominal CT, the peritoneal fluid volume remained stable and he did not require paracentesis for relief from abdominal distension or discomfort.

## Discussion

RET-specific TKIs were developed to decrease toxicity, increase efficacy, and cross the blood-brain barrier when compared to multikinase inhibitors (MKIs) with anti-RET activity [9]. Selpercatinib and pralsetinib were the first drugs of this class to obtain approval from regulatory agencies in 2020 [1]. The most common related adverse events of selpercatinib are hypertension (48%) and elevated transaminases (60-61%) [1]. Among less common toxicities, chylous effusions have been reported in up to 7% of patients receiving RET-selective TKIs across multicenter studies [5].

CA is a type of ascites characterized by its milky appearance and high concentration of triglycerides (over 200 mg/dL) [7]. Its incidence is very rare and can derive from multiple causes, with most cases of CA in developed countries being associated with malignancy and cirrhosis [10]. In the context of targeted therapies, isolated reports and retrospective analyses have identified associations between selective RET TKIs, particularly selpercatinib, and chylous effusions [11]. A retrospective multicenter study by Kalchiem-Dekel et al. [5] analyzed 7517 patients treated with either multikinase or selective RET TKIs. Chylous effusions (pleural or peritoneal or both) occurred in 22 patients, predominantly with selpercatinib (7%), followed by agerafenib (4%), cabozantinib (0.3%), and lenvatinib (0.02%), with a total of 10 patients presenting with CA. 

Considering our patient's surgical history, post-surgical lymphatic disruption was initially suspected but ultimately excluded due to the absence of surgical complications and no evidence of pleural effusion on follow-up CT scans performed prior to the initiation of selpercatinib. Lymphangiography was not performed in this patient. From literature, when performed it did not reveal a chyle leak, suggesting a mechanism unrelated to overt lymphatic disruption [5]. Although transdiaphragmatic fluid translocation secondary to suspected pleural metastases was considered, the patient never developed pleural effusion. The possibility of ascites caused by peritoneal implants was also ruled out, given the lack of peritoneal metastases on imaging and the absence of malignant cells in the peritoneal fluid analysis. 

Given the temporal association between the introduction of selpercatinib and the onset of CA, alongside exclusion of alternative causes and corroborating reports in the literature, the medical team concluded that the CA was an adverse effect of selpercatinib treatment. The underlying pathophysiological mechanism of these events, however, remains to be explained. Potential hypotheses include functional lymphatic endothelial changes or microvascular permeability alterations rather than a simple mechanical obstruction or RET inhibition.

Management guidance is scarce but includes a high-protein diet combined with restriction of long-chain triglycerides (favor medium-chain triglycerides). If necessary, pharmacological interventions such as somatostatin analogs or other agents such as orlistat and etilefrine can be used to increase vascular resistance, reducing lymphatic system flow or by inhibition of fat reabsorption in the bowels [7,10]. Dose reduction of TKIs was previously reported to not correlate with the rate of chylous effusion reaccumulation [5], but was an effective measure in this patient when associated with dietary management. In the event of CTCAE grade 3 or 4 reactions, withholding the drug until recovery to baseline is advised [8], which was not the case in this patient.

## Conclusions

TKIs have significantly advanced the treatment of RET fusion-positive NSCLC, but close monitoring for adverse effects remains essential. We present a case of selpercatinib-associated CA, a rare but notable complication. In this patient, dose reduction along with dietary modifications appeared to help manage symptoms without compromising disease stability. Although dose reduction has not been widely reported as a standard approach for managing peritoneal fluid accumulation with selpercatinib, this case suggests it may be a feasible option in selected patients. Further studies are needed to establish optimal management strategies for such adverse events.
